# Additional Diagnostic Value of Four-View Radiography in Acute Finger Trauma: A Retrospective Single-Center Diagnostic Cohort Study

**DOI:** 10.3390/jcm15145439

**Published:** 2026-07-11

**Authors:** Woo Young Jung, Dongkyung Seo

**Affiliations:** 1 Department of Orthopedic Surgery, Ayase Ekimae Orthopedic and Internal Medicine Clinic, Tokyo 120-0005, Japan; 2 Department of Plastic and Reconstructive Surgery, Faculty of Medicine and Graduate School of Medicine, Hokkaido University, Sapporo 060-8638, Hokkaido, Japan

**Keywords:** finger trauma, phalangeal fracture, radiography, oblique view, reverse oblique view, diagnostic yield, range of motion

## Abstract

**Background/Objectives:** Acute finger trauma is frequently encountered in outpatient orthopedic practice, and accurate initial radiographic diagnosis is essential for treatment planning. Standard two-view radiography, consisting of posteroanterior and lateral views, may miss projection-dependent phalangeal fractures. This study evaluated the additional diagnostic value of oblique and reverse oblique radiographs in acute finger trauma. **Methods:** This retrospective single-center diagnostic cohort study included patients with acute finger trauma who underwent four-view radiography at the initial visit. Images were reviewed in a two-step manner: first using standard two-view assessment and then after adding oblique and reverse oblique views. The primary outcome was the incremental diagnostic yield of additional views among final fracture cases. **Results:** Among 282 included episodes, 157 fractures and 125 non-fracture injuries were identified. Of the 157 fractures, 136 were detected on standard two-view assessment, whereas 21 were detected only after adding oblique and reverse oblique views, corresponding to an incremental diagnostic yield of 13.4% (95% confidence interval, 8.9–19.6%). Approximately one quarter of fractures were visible in only a single projection. Four-view-only fractures were more frequently located in the central digits. Residual range-of-motion limitation differed significantly among groups and was more frequent in both fracture groups than in the non-fracture group. **Conclusions:** Additional oblique and reverse oblique radiographs improved fracture detection in acute finger trauma. Four-view radiography may reduce projection-dependent underdetection of phalangeal fractures, particularly in injuries involving the central digits.

## 1. Introduction

Acute finger trauma is frequently encountered in outpatient orthopedic practice and emergency care. Phalangeal fractures are common upper-extremity injuries and may be associated with post-injury complications affecting finger and hand function [1]. Accurate detection of phalangeal fractures at the initial visit is important because the early treatment strategy, including the immobilization method, duration of fixation, referral decisions, and timing of rehabilitation, depends on the initial diagnosis [2,3,4,5]. In selected fracture patterns, operative fixation may be required, and contemporary fixation options for metacarpal and phalangeal fractures, including intramedullary screw fixation, remain under active discussion in the hand surgery literature [6]. Missed or underestimated fractures may contribute to delayed treatment, inappropriate immobilization, persistent pain, stiffness, and residual range-of-motion (ROM) limitation [3,5,7,8].

Standard radiographic assessment of hand and finger trauma commonly relies on two projections, typically a posteroanterior (PA) and a lateral view. Nevertheless, fracture visibility may depend strongly on projection. Obliquity of the fracture line, subtle cortical disruption, fragment overlap, and adjacent digit superimposition can make a fracture difficult to identify on standard views alone. Poor positioning or insufficient radiographic projections have also been associated with missed or misdiagnosed finger fractures [7].

Several authors and imaging recommendations have emphasized the value of additional projections in hand and distal extremity trauma [8,9,10]. However, the incremental diagnostic yield of adding both oblique and reverse oblique views to standard two-view radiography in acute finger trauma remains insufficiently quantified. In particular, limited evidence is available regarding how often fractures are visible only after additional views and whether such fractures are clinically negligible or associated with residual functional findings.

The purpose of this study was therefore to evaluate the additional diagnostic value of four-view radiography in acute finger trauma. Specifically, we quantified the incremental diagnostic yield of adding oblique and reverse oblique views to standard two-view assessment and examined the projection-dependent visibility and clinical characteristics of four-view-only fractures.

## 2. Materials and Methods

### 2.1. Study Design and Setting

This retrospective single-center observational diagnostic cohort study was conducted at Ayase Ekimae Orthopedic and Internal Medicine Clinic, Tokyo, Japan. The study used existing clinical and radiographic data from routine outpatient care. The study and reporting followed the Standards for Reporting Diagnostic Accuracy Studies (STARD) 2015 reporting guideline; STROBE principles for observational studies were also considered [11,12,13]. Because the study used existing clinical data generated during routine outpatient care, relevant RECORD principles were considered where applicable [14].

The study was conducted in accordance with the Declaration of Helsinki and approved by the JMA Ethics Review Committee (IRB number: 16000041; approval number: R8-2; approval date: 3 June 2026). Because this was a retrospective study using anonymized existing clinical data, informed consent was handled using an approved opt-out procedure.

### 2.2. Participants

Consecutive patients who presented to the clinic between 1 January 2023 and 28 February 2026 with acute finger trauma and underwent initial radiographic evaluation were screened. The clinic routinely performs four-view radiography for finger trauma unless there is a special reason to limit imaging, such as consideration of radiation exposure or other patient-specific circumstances. Therefore, four-view radiography was not selectively reserved for more severe injuries or cases with a higher pretest probability of fracture.

Eligible cases were acute finger trauma episodes in which initial four-view radiographs of the finger or hand were available. The analytic cohort focused on finger trauma and phalangeal fracture assessment; metacarpal injuries were not included in the final analytic cohort. Cases were classified as fracture or non-fracture according to the final investigator-based image classification. Patients who opted out after public disclosure of the study information were excluded.

### 2.3. Radiographic Protocol

Radiographic assessment consisted of four projections: PA, lateral, oblique, and reverse oblique views. The oblique and reverse oblique views were obtained at approximately 45° projection angles. All radiographs were obtained during routine clinical care at the initial visit. Radiographs were obtained using a diagnostic X-ray apparatus (KXO-15R; Toshiba Medical Manufacturing Co., Ltd., Otawara, Tochigi, Japan) and a digital radiography flat-panel detector (CALNEO Smart S47, DR-ID1200; FUJIFILM Corporation, Tokyo, Japan).

### 2.4. Image Review and Reference Standard

Images were reviewed in a two-step manner. First, only the PA and lateral views were assessed, and the presence or absence of fracture was recorded. Second, the oblique and reverse oblique views were added, and the presence or absence of fracture was reassessed.

During image review, personal identifiers, the treating physician’s diagnosis, clinical course, follow-up radiographs, and medical record descriptions were not reviewed. When the final investigator had also been the treating physician at the initial visit, image review was performed after an interval of at least two months.

All images were reviewed on the clinic’s PACS workstation using SYNAPSE 5, version 5.5.000V5.1 (FUJIFILM Medical Systems U.S.A., Inc., Stamford, CT, USA), and digital magnification was used when necessary for detailed assessment of cortical continuity, fracture line visibility, and fragment displacement. The final reference classification was image-based and categorized as fracture present or fracture absent.

No indeterminate result category was used; all cases in the final analytic cohort were classified as fracture present or fracture absent.

### 2.5. Outcomes

The primary outcome was the incremental diagnostic yield of additional oblique and reverse oblique views among final fracture cases. The denominator was the number of final fracture cases, and the numerator was the number of fractures not identified on standard two-view assessment but identified after adding oblique and reverse oblique views.

Secondary outcomes included the distribution of fracture visibility across the four projections, the proportion of fractures visible in only a single projection, clinical characteristics of fractures detected only after four-view assessment, residual ROM limitation at the final visit, follow-up duration, and agreement between the treating physician’s initial diagnosis and the final investigator-based classification.

### 2.6. Residual Range-of-Motion Assessment

Residual ROM limitation was assessed at the final visit using available medical record documentation. Absence of flexion limitation was defined as a fingertip-to-palm distance of 0 mm. Absence of extension limitation was determined visually when no apparent extension deficit was observed compared with the adjacent or contralateral digits. Cases in which residual ROM limitation could not be determined from the available medical records were treated as missing and were not included in the denominator of the ROM-specific analysis.

### 2.7. Statistical Analysis

Categorical variables are presented as numbers and percentages. Continuous variables are presented as means with standard deviations or medians with interquartile ranges, as appropriate. The incremental diagnostic yield and other proportions were reported with 95% confidence intervals (CIs) when applicable. Group comparisons for categorical variables were performed using the chi-square test or Fisher exact test, as appropriate. Continuous variables were compared using the Mann–Whitney U test or Kruskal–Wallis test, as appropriate.

The distribution of involved digits was compared between the two-view-detected fracture and four-view-only fracture groups. Central digits were defined as the second to fourth digits, and border digits were defined as the thumb and little finger. Because the four-view-only outcome included 21 events, predictive analyses were interpreted cautiously and model complexity was limited in accordance with recommendations for regression modeling with sparse events [15].

Diagnostic performance of the initial treating physician’s diagnosis was evaluated using the final investigator-based image classification as the reference standard. Sensitivity, specificity, positive predictive value, negative predictive value, accuracy, and Cohen kappa were calculated [16,17]. Follow-up duration was analyzed using the Kaplan–Meier method, and the overall difference among groups was assessed using the log-rank test. Pairwise log-rank comparisons were not performed in the initial analysis because follow-up duration was considered a secondary descriptive indicator of clinical course rather than a direct measure of fracture severity or treatment success. A two-sided *p*-value < 0.05 was considered statistically significant. All statistical analyses were performed using R version 4.5.2 (R Foundation for Statistical Computing, Vienna, Austria). No formal sample size calculation was performed; all eligible episodes during the predefined study period were included.

## 3. Results

### 3.1. Study Population and Diagnostic Classification

**Figure 1:**
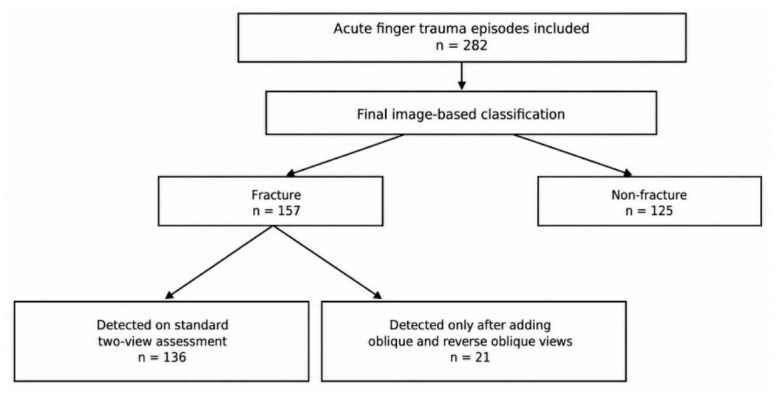
Study flow diagram. A total of 282 acute finger trauma episodes were included. Final image-based classification identified 157 fracture cases and 125 non-fracture cases. Among fracture cases, 136 were detected on standard two-view assessment, whereas 21 were four-view-only fractures.

A total of 282 eligible episodes of acute finger trauma were included in the final analysis. The median age of the study population was 16 years (interquartile range [IQR], 12–41 years; mean age ± SD, 26.1 ± 20.0 years). Of the 282 episodes, 127 (45.0%) occurred in males and 155 (55.0%) in females. The right hand was involved in 134 episodes (47.5%) and the left hand in 148 episodes (52.5%). Regarding the affected digits, the little finger was most frequently injured (84 episodes, 29.8%), followed by the index finger (52, 18.4%), ring finger (52, 18.4%), thumb (47, 16.7%), and middle finger (47, 16.7%). The median interval from injury to the initial clinic visit was 1 day (IQR, 0–2 days). Final image-based classification identified 157 fracture cases and 125 non-fracture cases (Figure 1). Among the 157 fracture cases, 136 fractures were detected on standard two-view assessment, whereas 21 fractures were detected only after adding oblique and reverse oblique views.

### 3.2. Incremental Diagnostic Yield of Additional Views

Among the 157 final fracture cases, 136 fractures were identified on standard two-view assessment. The remaining 21 were four-view-only fractures, corresponding to an incremental diagnostic yield of 13.4% (21/157; 95% CI, 8.9–19.6%) (Table 1).

### 3.3. Projection-Specific Fracture Visibility

**Figure 2:**
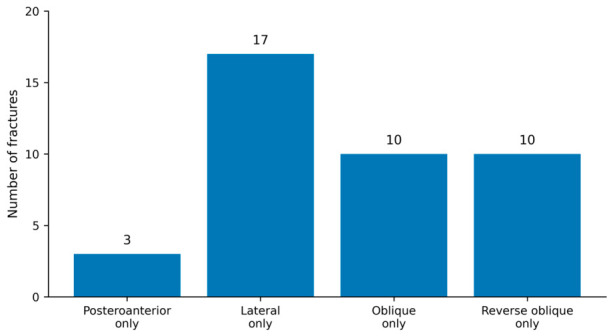
Fractures visible in only a single projection. Among 157 final fracture cases, 40 fractures were visible in only one projection: 3 on the PA view, 17 on the lateral view, 10 on the oblique view, and 10 on the reverse oblique view.

Among the 157 final fracture cases, 40 fractures (25.5%; 95% CI, 19.3–32.8%) were visible in only a single projection. These included 3 fractures visible only on the PA view, 17 only on the lateral view, 10 only on the oblique view, and 10 only on the reverse oblique view (Figure 2). Thus, approximately one quarter of fractures showed single-projection-dependent visibility.

### 3.4. Characteristics of Four-View-Only Fractures

The distribution of involved digits differed between the two-view-detected fracture and four-view-only fracture groups (*p* = 0.014). Four-view-only fractures were more frequently observed in the central digits, defined as the second to fourth digits, than in the border digits, defined as the thumb and little finger (Table 2). No significant association was observed between articular involvement and the need for four-view imaging (*p* = 0.48). Because the four-view-only fracture group included only 21 cases, the observed digit-distribution difference should be interpreted as an exploratory subgroup finding with limited precision.

### 3.5. Residual Range-of-Motion Limitation and Follow-Up Duration

ROM information at the final visit was available for 121 two-view-detected fracture cases, 19 four-view-only fracture cases, and 102 non-fracture cases. Residual ROM limitation differed significantly across the two-view-detected fracture, four-view-only fracture, and non-fracture groups (*p* = 0.027) (Table 3). Residual ROM limitation was observed in 36/121 two-view-detected fracture cases (29.8%), 8/19 four-view-only fracture cases (42.1%), and 18/102 non-fracture cases (17.6%). Fracture cases overall showed a higher rate of residual ROM limitation than non-fracture cases (44/140, 31.4% vs. 18/102, 17.6%; *p* = 0.015). The four-view-only fracture group also showed a higher rate of residual ROM limitation than the non-fracture group (42.1% vs. 17.6%; *p* = 0.030), whereas the difference between the two-view-detected fracture group and the four-view-only fracture group was not statistically significant. Given the small number of four-view-only fractures and the limited number of ROM-evaluable cases, the absence of a statistically significant difference between the two fracture groups should be interpreted cautiously.

Follow-up duration differed significantly across the two-view-detected fracture, four-view-only fracture, and non-fracture groups. Median follow-up duration was 36 days, 22 days, and 13 days, respectively. Kaplan–Meier analysis showed an overall difference among the three groups (log-rank *p* < 0.001).

### 3.6. Agreement Between Initial and Final Diagnoses

The initial treating physician and final investigator diagnoses showed high agreement. Among 282 episodes, 147 were classified as fracture by both assessments, 122 were classified as non-fracture by both assessments, 10 were classified as fracture only by the final investigator assessment, and 3 were classified as fracture only by the initial treating physician. Diagnostic performance of the initial treating physician diagnosis is shown in Table 4. Cohen kappa was 0.91, indicating almost perfect agreement.

### 3.7. Adverse Events

No adverse events related to routine radiographic assessment were identified in the available clinical records.

## 4. Discussion

The principal finding of this study was that additional oblique and reverse oblique views identified 13.4% of phalangeal fractures that were not detected on standard two-view radiographic assessment. In addition, approximately one quarter of fractures were visible in only a single projection. These findings demonstrate the projection-dependent nature of phalangeal fracture diagnosis and support the diagnostic value of four-view radiography in acute finger trauma.

The incremental diagnostic yield observed in this study is clinically relevant. A 13.4% additional detection rate means that relying on standard two-view assessment alone may leave a non-negligible subset of fractures undetected on initial assessment. This is consistent with prior work emphasizing that hand and finger fractures can be missed on poorly positioned or insufficient radiographs and that additional views may improve assessment of hand trauma [7,8,9,10]. Unlike previous studies that primarily discussed the need for additional radiographic views at a guideline or quality-improvement level, the present study quantified the incremental yield of adding oblique and reverse oblique views in a consecutive outpatient cohort.

An important secondary finding was the concentration of four-view-only fractures in the central digits. The second to fourth digits are located between adjacent rays and may be more affected by overlap on standard projections. Additional oblique and reverse oblique views may therefore be particularly useful for separating overlapping structures and clarifying subtle cortical disruption in these digits [5,7]. The absence of a significant association between articular involvement and four-view-only detection suggests that the need for additional views was not limited to intra-articular fracture patterns.

Because the median age was 16 years, pediatric and skeletally near-mature patients represented an important component of the cohort. Fracture visibility and healing patterns may differ in this age group because of open physes, incomplete ossification, and age-related fracture morphology. Although age-specific subgroup analyses were not performed, the findings support the usefulness of additional oblique and reverse oblique views for detecting projection-dependent phalangeal fractures in this population.

The single-projection-only analysis further supports the complementary role of multiple projections. Lateral-only fractures were the most frequent among single-projection-only cases, but a substantial number of fractures were visible only on oblique or reverse oblique views. These results indicate that each projection contributes distinct diagnostic information and that fracture visibility cannot be assumed from any single standard view.

The ROM findings suggest that four-view-only fractures were not merely trivial radiographic findings [3,4,5]. Residual ROM limitation was more frequent in fracture cases than in non-fracture cases, and the four-view-only fracture group showed a significantly higher rate of ROM limitation than the non-fracture group. However, the difference between the two fracture groups was not statistically significant. Therefore, four-view-only fractures should be interpreted as clinically relevant injuries, but the present data do not demonstrate that they are more severe than fractures visible on standard two-view assessment.

Follow-up duration also differed among the groups, with the longest median duration in the two-view-detected fracture group and the shortest duration in the non-fracture group. This finding should not be interpreted as a direct measure of fracture severity or treatment success, because outpatient follow-up may be influenced by symptom improvement, patient preference, physician-directed follow-up policy, and discontinuation of visits. Nevertheless, the longer follow-up observed in fracture groups is consistent with the clinical need for continued monitoring after confirmed fracture diagnosis.

The diagnostic agreement between the treating physician and the final investigator assessment was high, with a Cohen kappa of 0.91. This finding supports the reliability of routine clinical diagnosis in this cohort. At the same time, investigator-only fractures were still observed, underscoring the importance of systematic image review and adequate projections when evaluating acute finger trauma.

This study has several limitations. First, it was retrospective and conducted at a single clinic, which may limit generalizability. Second, the study reflects a clinical setting in which four-view radiography is routinely performed for finger trauma; therefore, the findings may not directly apply to institutions where additional views are obtained only selectively. Third, the final reference standard was based on investigator image review rather than independent multi-reader adjudication or advanced imaging for all cases. Therefore, this study should not be interpreted as a conventional diagnostic accuracy study against an external gold standard, but rather as a quantification of projection-dependent improvement in fracture detection by the same reader. The observed 13.4% incremental yield represents fractures that were not detected on the initial two-view assessment but became identifiable after additional projections were available, rather than an absolute estimate of fractures missed against an independent reference standard. This distinction is important because additional projections may reduce under-calling of subtle projection-dependent fractures, while very subtle cortical irregularities may still carry some risk of over-calling in the absence of advanced imaging or independent adjudication. To mitigate this limitation, a staged review procedure was used, clinical information was not reviewed during image assessment, images were assessed on a PACS workstation with digital magnification when necessary, and cases initially managed by the same investigator were reviewed after an interval of at least two months. Fourth, ROM outcomes were extracted from clinical records, and cases without sufficient documentation were treated as missing for ROM-specific analysis. Finally, the number of four-view-only fractures was modest, which limits the precision of subgroup, ROM-related, and predictive analyses; therefore, significant and non-significant subgroup comparisons should both be interpreted cautiously.

Despite these limitations, the study provides quantitative evidence supporting the additional diagnostic value of four-view radiography in acute finger trauma. The combination of incremental diagnostic yield, single-projection-only fracture visibility, central digit concentration, residual ROM findings, and high diagnostic agreement suggests that additional oblique and reverse oblique views can contribute meaningfully to the initial evaluation of phalangeal fractures.

## 5. Conclusions

Additional oblique and reverse oblique radiographs improved projection-dependent fracture detection in acute finger trauma by identifying 13.4% of fractures that were not detected on standard two-view assessment. Approximately one quarter of fractures were visible in only a single projection, underscoring the projection-dependent nature of phalangeal fracture diagnosis. Four-view-only fractures were concentrated in the central digits and were associated with residual ROM limitation compared with non-fracture cases. These findings support four-view radiography as a clinically useful diagnostic approach for acute finger trauma, particularly when phalangeal fracture is suspected.

## Figures and Tables

**Table 1:** Detection categories and incremental diagnostic yield among final fracture cases.

Assessment Category	*n*/*N*	Percentage	95% CI
Detected on standard two-view assessment	136/157	86.6%	80.4–91.1%
Four-view-only fractures	21/157	13.4%	8.9–19.6%

The denominator is the number of cases finally classified as fractures by the final investigator-based image classification.

**Table 2:** Distribution of fracture detection categories according to involved digit.

**A. Individual Digit Distribution**		
**Digit**	**Two-view-detected fractures (*n* = 136)**	**Four-view-only fractures (*n* = 21)**
Thumb	19	1
Index finger	22	8
Middle finger	21	5
Ring finger	27	6
Little finger	47	1
**B. Central versus border digit classification**		
**Digit category**	**Two-view-detected fractures (*n* = 136)**	**Four-view-only fractures (*n* = 21)**
Border digits (thumb and little finger)	66	2
Central digits (index, middle, and ring fingers)	70	19

The overall *p*-value for individual digit distribution was *p* = 0.014. Central digits were defined as the second to fourth digits; border digits were defined as the thumb and little finger. Comparisons involving four-view-only fractures should be interpreted cautiously because of the limited subgroup size.

**Table 3:** Residual range-of-motion limitation at the final visit.

Group	ROM Limitation Present	ROM Limitation Absent	Rate
Two-view-detected fracture group	36	85	36/121 (29.8%)
Four-view-only fracture group	8	11	8/19 (42.1%)
Non-fracture group	18	84	18/102 (17.6%)

Cases in which residual ROM limitation could not be determined from the available medical records were treated as missing and were not included in the denominator of the ROM-specific analysis.

**Table 4:** Diagnostic performance of the initial treating physician diagnosis compared with final investigator assessment.

Measure	Value
True positive/true negative/false negative/false positive	147/122/10/3
Sensitivity	93.6% (147/157; 95% CI, 88.6–96.8%)
Specificity	97.6% (122/125; 95% CI, 93.2–99.5%)
Positive predictive value	98.0% (147/150; 95% CI, 94.3–99.6%)
Negative predictive value	92.4% (122/132; 95% CI, 86.5–96.0%)
Accuracy	95.4% (269/282)
Cohen kappa	0.91

The final investigator-based image classification was used as the reference standard.

## Data Availability

The data presented in this study are available on request from the corresponding author due to privacy and ethical restrictions.

## Abbreviations

Abbreviation	Definition
CI	Confidence interval
IQR	Interquartile range
IRB	Institutional review board
PA	Posteroanterior
PACS	Picture archiving and communication system
ROM	Range of motion
SD	Standard deviation
